# The Aural Complications of Influenza and Their Treatment

**Published:** 1908-09

**Authors:** Harold F. Mole

**Affiliations:** Assistant Surgeon and Surgeon in Charge of the Aural Department, Bristol Royal Infirmary


### THE AURAL COMPLICATIONS OF INFLUENZA AND THEIR TREATMENT.

BY

Harold F. Mole, F.R.C.S.,

*Assistant Surgeon and Surgeon in Charge of the Aural Department, Bristol Royal Infirmary.*

---

Now that the influenza season is again approaching, it seems a not unfitting time to call attention, in a very brief way, to the various ear troubles it may give rise to, especially as these form a fairly frequent complication of this affection. Amongst adults it is one of the commonest causes of suppurative otitis, and probably accounts for more of this trouble in children than is generally supposed ; and particularly is this the case in those suffering from adenoids. Common catarrhs, adenoids, measles and scarlet fever are, however, more frequent causes in children. Although there are some peculiarities in influenzal otitis to which attention must be called, there is, however, no very material difference in treatment between it and that due to other specific affections.

Influenza may set up acute otitis media by way of the Eustachian tube, which may be non-suppurative or suppurative, the latter being the more common.

With regard to the former, the symptoms are acute pain in the ear, with congestion of the drum, and probably blocked Eustachian tube and deafness. The treatment to be adopted will be that of antiseptic sprays or irrigations to the naso-pharynx, leeches to the front and back of the ear (if the symptoms are severe), instillation of a few drops of warmed carbolised glycerine into the meatus, and hot *dry* applications over the ear. A brisk aperient should also be administered. As the acute symptoms subside, the ear should be gently inflated by Politzerising or catheter—preferably the latter, as it is more accurate, and the force of the inflation can be better regulated.

The evidence of case histories shows that this acute non-suppurative otitis caused by influenza is not infrequently the precursor of chronic adhesive middle-ear catarrh. It is, therefore, of the utmost importance to treat it with the greatest care in the early stages, and not to let the patient pass from one's hands until cured.

Suppurative otitis media in influenza is often characterised by the extreme acuteness of the process. with ecchymosis, and the formation of hemorrhagic blebs on the drum and on the deeper parts of the meatus, and also by its great tendency to involve the mastoid process. Accompanying these appearances are intense pain of neuralgic character, and, of course, deafness, the latter often coming on more gradually than in other forms of otitis. Another peculiar feature is that this pain is not always immediately relieved when the drum has burst or has been incised, which is the case in other forms of suppurating ears. The early treatment of these cases should be the same as mentioned above in the non-suppurative variety. A careful watch must, however, be kept for signs of suppuration, and persisting intense pain or bulging drum should be treated by free incision of the membrane from top to bottom behind the handle of the malleus. This treatment is far better than allowing the drum to perforate, since it is followed by immediate drainage, and healing occurs more quickly than in the case of spontaneous perforation. As mentioned above, however, the pain may persist for a few days after the incision. The treatment after incision will depend upon the fancy of the surgeon, and the conditions under which it must be carried out. When the surgeon is able to carry out this himself, thorough cleansing and drying of the ear, and packing down to the perforation with a strip of moist antiseptic gauze as often as is necessary, is perhaps the best. This, however, is seldom possible, and when, as more often happens, the patient's friends have to carry out the treatment, I am in the habit of ordering irrigations (not syringing) of warm boric lotion, carried out frequently, a large quantity of lotion being used each time. The frequency with which this should be carried out will depend upon the amount of discharge. If the incision is free or the perforation large, it is often unnecessary to order any other treatment than this, as the discharge rapidly diminishes, and the perforation heals in a week or two or less. When, however, all the acute symptoms have passed, and the discharge is much diminished but the perforation is sluggish in healing, a little boric powder may be insufflated, or some glycerine of carbolic acid, 1 in 10, instilled after the ear has been cleansed and dried. When the perforation is spontaneous and small, and especially if it is at the apex of a nipple-like projection of the drum, it should be enlarged by incision ; but if this is not indicated, and the perforation is still small, the best plan is to instil peroxide of hydrogen (hydrozone and perhydrol are strong solutions of the same) for fifteen minutes ; mop dry or syringe, and then instil for a similar period a few drops of glycerine of carbolic acid, 1 in 10, or of glycerine of borax with two drams of absolute alcohol to the ounce. Irrigation and syringing are not of much value when the perforation is small ; they only cleanse the meatus. After the perforation has firmly healed it will probably be found necessary to carry out a course of inflations through the Eustachian tube for a time, to completely restore the hearing. If there are not, after three weeks of the above treatment, clear indications that the affection is coming to an end and the perforation closing, it must be assumed either that the antrum is not draining properly through the tympanum, or that the mastoid process is involved. Under these circumstances the antrum must be opened and drained for a time, and the tympanic cavity washed out by this route.

As before mentioned, one of the peculiarities of influenzal otitis is its tendency to involve the mastoid process. This involvement may take place before there is any perforation of the drum, or indeed, although rarely, without there being any discharge from the meatus. It may involve the mastoid cells without there being any obvious communication with the tympanum, and it may cause rapid destruction of the bone, early exposing the lateral sinus or dura mater, and giving rise to any of the ordinary intracranial complications. The diagnosis of influenzal mastoiditis is not always easy, and it must be remembered that pain and tenderness over the mastoid at the *commencement* of the ear trouble will often subside under active antiphlogistic treatment if there is a free opening in the drum. Temperature is very little guide, as it is often subnormal, with the mastoid full of pus and carious bone. A persisting tenderness after the first few days with no abatement in the discharge, with or without swelling, and œdema over the mastoid process, indicates operation without delay. In most cases it will be found necessary to remove the whole of the outer cortex of the mastoid from apex to antrum. Should a specially tender spot be noticed, this may be explored first, for the abscess may then be localised. I have seen cases in which the mastoid cells were filled with pus and carious and necrotic bone, and the antrum unaffected, and apparently unconnected with them. After removal of the cortex, all diseased bone should be thoroughly scraped away, and the cavity packed with gauze. It will, of course, be understood that in the acute cases I have been discussing the ossicles are not interfered with nor the drum, unless it is necessary to enlarge an opening in the latter. In this short sketch the intracranial complications cannot be discussed.

Finally, there are those cases of post-influenzal deafness which on examination are found to be of nervous origin. They are probably due to a neuritis of the auditory nerve, though some are supposed to be caused by changes in the labyrinth. There is not infrequently severe tinnitus with them. In this case the treatment consists in the administration of strychnine, either hypodermically or by the mouth. The treatment must be persisted in for some weeks, and the quantity increased gradually until a full dose is reached.

